# Condensation and dissolution of nematic droplets in dispersions of colloidal rods with thermo–sensitive depletants

**DOI:** 10.1038/srep18432

**Published:** 2015-12-14

**Authors:** Anna Modlińska, Ahmed M. Alsayed, Thomas Gibaud

**Affiliations:** 1 Labaroire de physique, CNRS/UMR 5672, Ecole Normale Supérieure de Lyon – Université de Lyon, 46 allée d’Italie, 69007 Lyon, France; 2 Faculty of Technical Physics, Poznan University of Technology, ul. Piotrowo 3, 60-965 Poznań, Poland; 3 Complex Assemblies of Soft Matter (COMPASS), Solvay-CNRS-UPenn UMI 3254, Bristol, Pennsylvania 19007, USA

## Abstract

Nematic droplets are droplets composed of elongated molecules that tend to point in the same direction but do not have any positional order. Such droplets are well known to adopt a spindle shape called tactoid. How such droplets condensate or melt and how the orientational symmetry is broken remains however unclear. Here we use a colloidal system composed of filamentous viruses as model rod–like colloids and pnipam microgel particles to induce thermo–sensitive depletion attraction between the rods. Microscopy experiments coupled to particle tracking reveal that the condensation of a nematic droplet is preceded by the formation of a new phase, an isotropic droplet. As the viruses constitute an excellent experimental realization of hard rods, it follows that the phenomenology we describe should be relevant to diverse micro- and nano-sized rods that interact through excluded volume interactions. This transition between isotropic and nematic droplets provides a new and reversible pathway to break the symmetry and order colloidal rods within a droplet with an external stimulus, and could constitute a benchmark experiment for a variety of technologies relying on reconfigurable control of rods.

Reconfigurable self-assembly of rod-like molecules leads both to a better fundamental understanding of phase transition^1^,^2^ and a variety of technologies which rely and urge for switchable molecular structures such as electro-optical modulators, optical switches, light shutters^3^,^4^ or biosensors^5^. Mixture of monodisperse micron-long filamentous bacteriophages and non-adsorbing polymers self- assemble into a myriad of structures ranging from isotropic, nematic, twisted ribbons, colloidal membranes and colloidal rafts^1^,^6^,^7^,^8^. Here we focus on the isotropic nematic phase transition. Nematic droplets are droplets composed of elongated molecules that tend to point in the same direction but do not have any positional order. Such droplets are well known to adopt a spindle shape called tactoid^9^,^10^,^11^. The shapes results from the molecules orientation and the interplay between the interfacial tension and the splay and bend elastic constants of the nematic domain^12^. Yet choosing and controlling upon external signaling a reversible pathway to form and dissolve tactoids remains a challenge because it requires both to change the density and to break the symmetry.

The conventional route that leads to tactoid formation consists in increasing the density of a dispersion of rods in the isotropic state, a state with neither translational nor rotational order. As Onsager predicted in his pioneering work^13^, homogeneous dispersions of very elongated stiff particles with hard core repulsive interactions can become metastable or unstable and phase separate as its density increases: orientation fluctuations in the isotropic state drive concentration differences, resulting in a phase separation of the rods in an isotropic state and a nematic state. Nematic droplets formed during this transition are transient. They are the fingerprint of early stage of phase separation. Indeed, they eventually coalesce to minimize the interface between the isotropic and the nematic phases to achieve the thermodynamically stable state: two homogeneous phases, the isotropic and nematic phase separated by a single interface. This transition has been observed in many experimental systems, tobacco mosaic viruses (TMV)^14^, fd–viruses^15^, F–actin^16^, bohemite rods^17^, carbon nanotubes^18^,^19^,^20^, liquid crystal molecules^21^,^22^ and computer simulations^23^,^24^. This transition is purely entropic and counterintuitive^25^: how can a hard-rod system gain entropy by going from a disordered fluid phase to an orientationally ordered phase? The loss in entropy due to the orientation ordering in the nematic phase is over compensated by the increase in translational entropy of the system: the available space for any one rod increases as the rods become more aligned. This conventional pathway is however inadequate to study tactoid formation; it necessitates a concentration quench which is tricky to realize. Following this problem, Lettinga *et al.*^26^ used shear to form an isotropic state from a nematic state. This route to tactoid formation remains however kinetically driven as it starts from an unstable state and is therefore hard to implement on technological devises.

Those route to tactoids formation are static: once assembled, it is difficult to reconfigure the droplet. To circumvent this issue we have engineered a colloidal system where we can continuously tune with temperature the attraction between the rods to condensate tactoids in a reversible and quasi static way starting from an equilibrium state. The colloidal dispersion consists of a dilute aqueous suspension of semi–flexible and monodisperse filamentous viruses M13K07. Utilization of colloidal size viruses allow for their visualization with single particle resolution and provide an excellent experimental tool to study such transitions at the molecular level^27^,^28^,^29^,^30^,^31^. We add thermo–sensitive and non-adsorbing pnipam microgel particles^32^ to the otherwise repulsive virus dispersion to induce an effective attraction controlled externally by temperature. This method allows to navigate in the phase diagram of rods dispersions in a continuous way. In particular, we show that the condensation of a nematic droplet is preceded by the formation of an isotropic droplet thus providing a new and reversible pathway to order colloidal rods within a droplet with an external stimulus. As the viruses used in our studies are an excellent experimental realization of hard rods^15^, it follows that the phenomenology we describe should be relevant to diverse micro- and nano-sized rods that interact through excluded volume interactions.

In this paper, first, we present the characteristics of our system, namely colloidal rods dispersed in buffer with thermo–responsive microgel particles. Then, we characterize the morphology of the nematic droplet during its formation and dissolution. Phase contrast and cross–polarization microscopy visualization reveals that upon increasing the attraction between the rods, first, the rods condensate into an isotropic and spherical droplet which then grow and finally evolve into a tactoid with nematic order. The dense isotropic spherical droplet is shown for the first time and is the key step that allows to break the symmetry. Finally, direct visualization of rods orientation with single particle resolution fluorescence microscopy allows us to track the motions of individual rods and to determine the translational and orientation dynamics of the rods and the order parameter.

## Results and Discussion

### Dispersions of colloidal rods and pnipam microgel particles

As a model semi–flexible rod–like particles we use the filamentous bacteriophage M13K07 with a contour length of *l*_*V*_ = 1.2 *μ*m, a diameter of *d*_*V*_ = 6.6 nm and a persistence length of 2.8 *μ*m^33^. M13K07 viruses are synthesized using standard biological protocols^33^, see Methods. Monodisperse viruses are dispersed at c_*V*_ = 0.3 mg/mL in 20 mM TRIS buffer at pH = 8.0 and 100 mM NaCl to screen the electrostatic charges. We chose a very low virus concentration to focus on the effect of the depletents and minimalize the effect of critical fluctuations which, in the absence of depletants, are maximal in the vicinity of the I-N lower critical concentration ≈10 mg/mL. Such system display a very rich phase diagram^1^,^6^,^7^,^8^.

**Figure 1:**
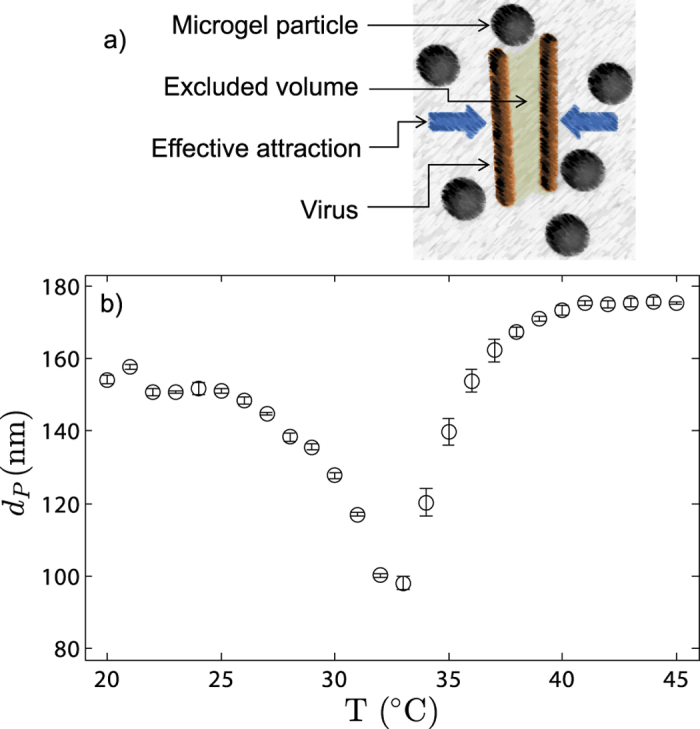
The addition of pnipam microgel particles to the virus dispersion mediates an effective thermo–sensitive attraction between the colloidal rods. (**a**) Schematic of the depletion attraction mechanism. Rods tend to condensate and align so that the excluded volume Δ*V* for the microgel particles is maximized. (**b**) Temperature dependence of the hydrodynamic diameter of the pnipam microgel dispersion diluted in buffer to the concentration of *c*_*P*_ = 0.3 mg/ml measured by means of DLS. Each measurement is performed during 1 min and repeated 5 times after a waiting time of 10 min. From 20 °C to 33 °C, the diameter of the microgel particle decreases. Due to high salt concentration, above 33 °C, the microgel particles start to aggregate and the apparent diameter increases.

Thermo–responsive and non–adsorbing pnipam microgel particles are synthesized according to a standard protocol^34^, see Methods. The pnipam microgel particles are dissolved in the buffer at c_*P*_ = 30 mg/mL and mixed with the viruses at c_*V*_ = 0.3 mg/mL. The microgel particles act as a depletant and create an effective interaction between the viruses^35^. The depletion interaction is entropic in nature. For rod–like particles, the depletion attraction is anisotropic and tends to align the viruses^8^,^36^, Fig. 1a, and may trigger the *I* − *N* transition^24^,^31^,^37^,^38^. The use of pnipam microgel particles makes the depletion interaction temperature dependent. When rods are dispersed in the presence of non adsorbing pnipam microgel particles, each rod is surrounded by an exclusion zone depleted of microgel particles with a thickness corresponding to the diameter, *d*_*P*_, the microgel particles. When the surface of two parallel rods approach to a distance closer than *d*_*P*_ the two exclusion zones overlap by Δ*V* resulting in an osmotic pressure imbalance that drives the rods together as illustrated in Fig. 1a. The contact attraction energy between two rods is *U*(*n*_*P*_, *d*_*P*_) = −*P*Δ*V*^39^. The osmotic pressure is *P* = *n*_*P*_*k*_*B*_*T*, assuming the pnipam microgel particles behave as an ideal gas with a number density *n*_*P*_. The excluded volume is 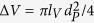, assuming *d*_*P*_ » *d*_*V*_. In the regime going from 20 to 33 °C, *n*_*P*_ is constant but *d*_*P*_ decreases with temperature and therefore so does the strength of the attraction. In the second regime, above 33 °C, both *P* and Δ*V* vary significantly. The number density that matters now is the one of the aggregates and the osmotic pressure rewrittes as *P* = *n*_*P*_(*d*_*P*_(33 °C)/*d*_*P*_)^3^*k*_*B*_*T*, where *d*_*P*_ is now the diameter of the pnipam microgel particles aggregate and *d*_*P*_(33 °C) is the diameter of the pnipam microgel particles at 33 °C, just before aggregation. *U* = −*nk*_*B*_*T*(*d*_*P*_(33 °C))^3^*πl*_*V*_/(4*d*_*P*_) and the strength of the attraction continues to decrease as *T* increases. To conclude, this qualitative estimation of the attractive potential shows that the strength of the attraction decreases through the entire range of temperatures. As *T* increases from 20 to 33 °C, *P* remains mostly constant and Δ*V* is the leading source that decreases the strength of the attraction. The range of the attraction decreases as *d*_*P*_, see Fig. 1b. Above 33 °C, *P* decreases more than Δ*V* increases which leads again to a decrease of the strength of the attraction with *T*. The range of the attraction increases as *d*_*P*_, see Fig. 1b.

### Condensation and melting of nematic droplets

**Figure 2:**
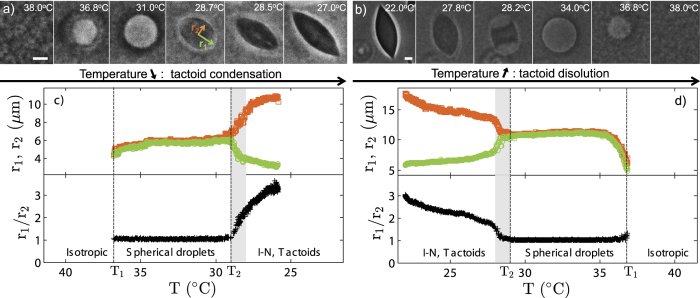
Condensation and dissolution of nematic droplets. (**a**,**b**) Phase contrast images of the condensation and dissolution of nematic droplets as a function of temperature *T* formed in the colloidal suspension of M13K07 viruses mixed with pnipam microgel particles at *c*_*P*_ = 30 mg/ml. As temperature is lowered the attraction increases. The rate of the quench is 0.09 °C/min, Supporting Information Fig. 1. (**c,d**) Measurements of the long and short semi-axis of the droplet, *r*_1_ and *r*_2_ as well as the ratio *r*_1_/*r*_2_ as a function of temperature. *T*_1_ is the temperature that separates the isotropic state from the spherical droplets regime. *T*_2_ is the temperature that separates the isotropic droplets regimes from the tactoids regime. Scale bars are 5 *μ*m.

**Figure 3:**
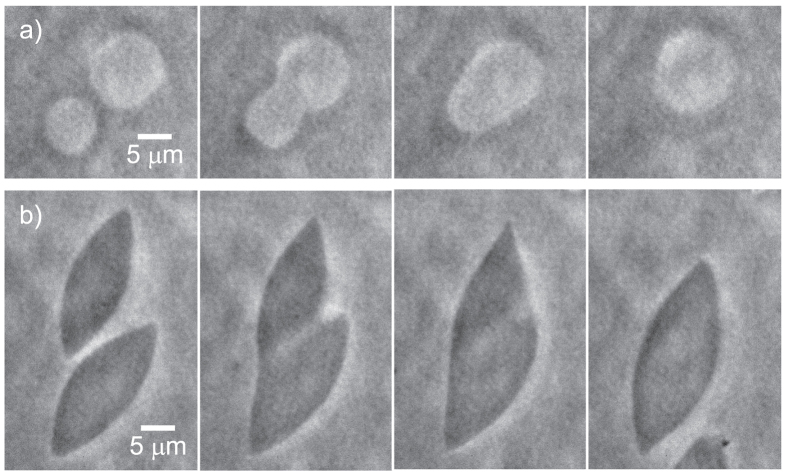
Coalescence processes. (**a**) Coalescence of two isotropic spherical droplets. (**b**) Coalescence of two tactoids.

Figure 2a and Supplementary Movie 1 show phase contrast imaging of a temperature quench from *T* = 40 °C to 22 °C of the aqueous dispersion of rods and pnipam microgel particles. First, at high temperatures, the dispersion is isotropic, the strength of the depletion interaction is small and the rods form a homogeneous state. As we decrease the temperature, the strength of the depletion interaction increases and we observe larger density fluctuations that eventually lead to the nucleation of spherical droplets at *T*_1_ = 36.8 °C. The fact that the droplets are contrasted with respect to the background proves that the refraction index of the droplet is different from the one of the background. The droplets are therefore more concentrated in viruses than the background but remain in an isotropic state; cross polarization imaging show no birefringence. The radius of the smallest droplets we could observe in our system is 4.0 ± 0.5 *μ*m. Then the radius and the contrast of the droplet increases. The edge of the droplet fluctuates a lot meaning a low surface tension. However compared to the bulk energy, the surface tension is not low enough to stabilize the droplets and the droplets coalesce (Fig. 3 and Supplementary Movie 1). At *T*_2_ = 28.6 °C, in grey area in Fig. 2a,b, the droplets morph into tactoids. Under crossed polarizers, the background fluid is isotropic and remains dark. In contrast tactoids show birefringence that confirms their liquid crystalline nature. The variation of the texture of the tactoids observed with the rotation of the polarizers (Supplementary, Fig. 2) is indicative of a bipolar director field^40^,^41^. Viruses form a nematic phase which director is aligned with the long axis of the tactoids. Close to the contour of the tactoid, the director field follows the shape of the tactoids. The fluctuations of the edge of the tactoid are much lower than the ones observed for the droplet regime, probably because of the lateral surface anchoring of the rods, and not surprisingly tactoids also coalesce, see Fig. 3 and Supplementary Movie 1.

Upon heating the sample, we observe a similar scenario (Fig. 2d): first tactoids in an isotropic background below *T*_2_, then isotropic droplets in an isotropic background and finally a homogeneous isotropic state above *T*_1_. Those transitions are reversible and quasi-static. All tactoids and isotropic droplets appear within ±0.5 °C of *T*_1_ and *T*_2_, Fig. 2c,d. The transition is independent of three cooling or heating rate we have tested: ~0.09, ~0.30 and ~1.35 °C/min. We however observe a slight dissymmetry in the evolution of *r*_1_ and *r*_2_ upon cooling (Fig. 2c) or heating (Fig. 2d). We attribute the dissymmetry to the initial size of the droplets. Indeed, starting from the isotropic state, isotropic droplets nucleate with rather small radius, about 4 *μ*m. On the contrary, starting from the I-N coexistence, the tactoids preexist and tend to coalesce. Finally, the morphogenesis of the tactoid into a droplet, at *T*_2_ = 28.6 °C in grey area in Fig. 2a,b, is much clearer during the heating process because the tacoids are much larger. This process is remarkable and is seemingly intrinsically 3D in nature. It looks like the point defects located at the extremity of the long semi-axis of the tactoid bend upward on themselves until they meet at the center of the droplet and merge to give shape to a spherical droplet.

### Shape of the nematic droplet

**Figure 4:**
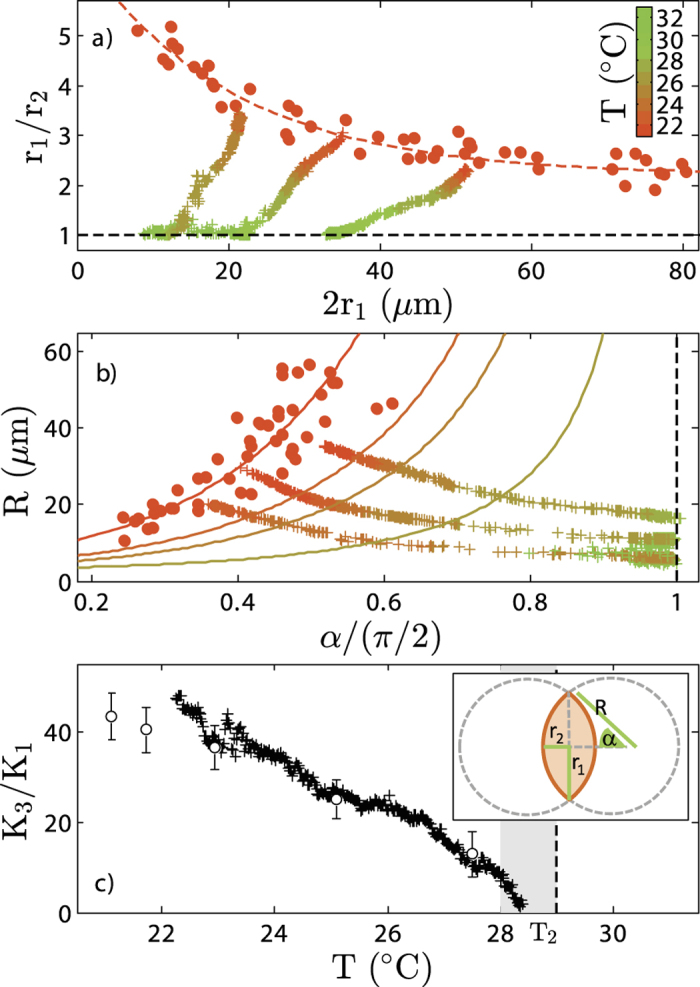
Macroscopic properties of the tactoids. (**a**) Aspect ratio as a function of droplets length at *T* = 22 °C measured from phase contrast images for 47 different tactoids. Dash lines are guide to the eye. + indicates the evolution (2*r*_1_, *r*_1_/*r*_2_) of three different tactoids as a function of temperature. (**b**) Radius *R* as a function of the angle *α* at *T* = 22 °C computed from (**a**). The colored + indicates the evolution (*α*, *R*) of three different tactoids as a function of temperature. Lines are fit to data. (**c**) Bend and splay elastic constants ratio obtained from the fitting procedure as a function of temperature. Circles are data obtained by fitting the geometrical properties of ~50 different tactoids at a given temperature. + are the average evolution of *K*_3_/*K*_1_ based on the fitting of the geometrical properties of three different tactoids. Inset: shape of the tactoid modelled as a three–dimensional droplet formed by the rotation of two overlapping circles.

The distinctive, elongated shape of the tactoids can be described in two ways. On the one hand, the nematic droplet can be characterized by the dimension of its long semi-axis, *r*_1_ and short semi-axis *r*_2_^42^,^43^. On the other hand, the tactoids can be modeled as a three–dimensional droplet formed of rotation of two overlapping circles. Detailed analysis provided by Kaznacheev *et al.*^11^,^44^ combines the dimensions of the tactoid with the radius of circles *R* and the angle *α* corresponding to half of the arc length of the overlapping region as sketch in the inset in Fig. 4 and gives the following relations:


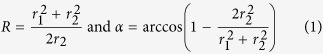


Both treatments presume a strong anchoring between the director and tactoid surface. The shape of nematic droplets results then from the minimization of the free energy where splay and bend elastic energies and the surface tension of the droplets compete with each other. Following those two approaches, we measure *r*_1_, *r*_2_, Figs 2c,d and 4a and compute *α* and *R* as a function of temperature, Fig. 4b. For transparency, we show the temperature dependences only for some of the tactoids. Figure 4a shows general features of the tactoids shape: smaller tactoids have higher aspect ratios and an increase of tactoid length results in two times smaller *r*_1_/*r*_2_ ratio. This trend is consistent with equilibrium tactoid shapes calculated on the basis of minimization of free energy and scaling theory presented in^42^ where only very high value of anchoring strength is taken into account. Scatter of the aspect ratios measured for droplets with similar length results from the fact that not all tactoids are located in the focal plane. Since tactoids shape resemble three–dimensional flat convex lenses their observation above the central cross–section gives smaller *r*_1_/*r*_2_ ratio. As shown in Fig. 4a, the aspect ratio of the tactoids *r*_1_/*r*_2_ shows that independently from the initial tactoid size all nematic droplets lose their anisotropic shape and tend to become circular isotropic droplets with an aspect ratio equal to one at *T*_2_.

*R* and *α* define the shape of the tacoids. Figure 4b shows some possibly trajectories of the melting/condensation process in the (*R*, *α*) space. Those trajectories explore a wide pathway for the tactoid to droplet transition: a slight change is *R* and *α* affect the trajectory of the melting/condensation process. It is therefore difficult to compare quantitatively single experiments such as the ones presented in Fig. 2c,d 11, we obtain *K*_1_/*σ*, *K*_3_/*σ* and the ratio *K*_3_/*K*_1_. *K*_1_ and *K*_3_ are the splay and bend elastic constants, respectively, and *σ* is the surface tension of the nematic–isotropic interface. For instance, at *T* = 21 °C, we obtained *K*_1_/*σ* = 1.9 ± 0.5 *μ*m, *K*_3_/*σ* = 85 ± 9 *μ*m and *K*_3_/*K*_1_ = 43 ± 15. The ratio *K*_3_/*K*_1_ is one order of magnitude higher as compared to typical thermotropic liquid crystals for which it does not exceed 3^45^. This, however, is consistent with values obtained for F–actin (≈54)^16^ or vanadium pentoxide (≈22)^11^. Such high values of *K*_3_/*K*_1_ at low temperatures reflect a high energy cost of bend with respect to splay. This is the fingerprint of (ultra)high aspect ratios of the viruses with respect to molecular liquid crystals which leads, at a macroscopic scale, to a strongly anisotropic shaped tactoid. As temperature increases, we observe that the tactoids become fatter and that the tip angle of the tactoids widens. This is consistent with the fit results: both *K*_1_/*σ* and *K*_3_/*σ* decreases, Fig. 4c. As we approach *T*_2_, *K*_1_/*σ* decreases and favors tactoids with wide tip angles; *K*_3_/*σ* also decreases and favors tactoid with high curvature. Ultimately, at *T*_2_, the tactoid becomes a spherical isotropic droplet with only a surface tension and a priori no elastic constants.

### Dynamics of individual rods within isotropic and nematic droplets

**Figure 5:**
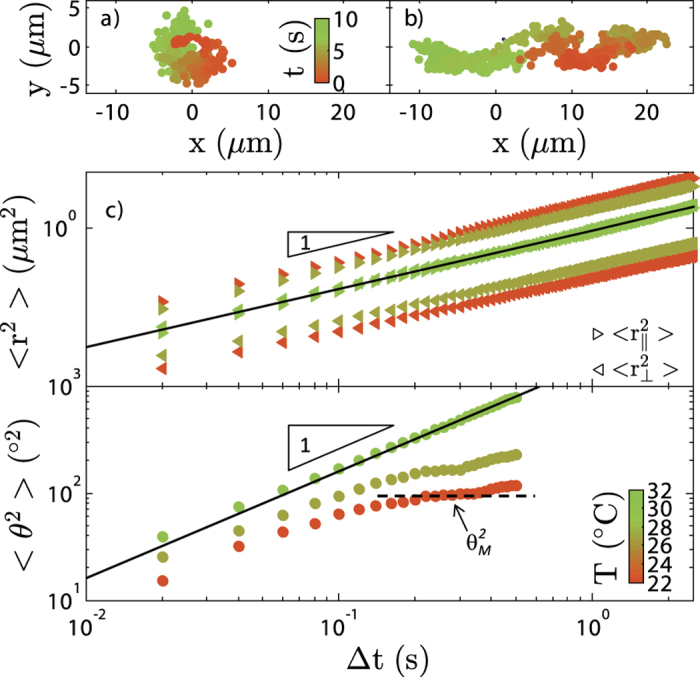
Local displacement of the rods within the nematic and spherical droplets. (**a**) Trajectory of a fluorescently labeled virus within the spherical droplet, see Supplementary Movie 3 and 4. (**b**) Trajectory of a fluorescently labeled virus within the tactoid. (**c**) Mean square displacement of viruses along 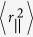 and perpendicular 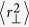 to the director as well as mean square displacement of the angle between the long axis of virus and tactoid director 〈*θ*^2^〉 presented at three different temperatures as one approaches the isotropic state from the nematic state.

During the *I* − *N* transition, not only do we observe modifications of the droplets shape but we also show that the dynamics and orientation of individual rods within the droplets changes. Figure 5a,b displays the trajectory of fluorescently labeled viruses in a background of unlabeled rods respectively for an isotropic spherical droplet and a tactoid. In the tactoid, the trajectory of the viruses is anisotropic. Viruses are moving mostly along the long axis indicating nematic order. In contrast, in the spherical droplet, the trajectory of the rods is isotropic which is characteristic of the isotropic state.

The mean square displacements as a function of time in the nematic region are plotted in Fig. 5c. The linear dependence of 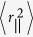 and 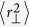 over the whole time range confirms the diffusive motion of the rod–like molecules within droplets. The diffusion along the director is one order of magnitude higher than the diffusion perpendicular to the director of the nematic phase which is consistent with data obtained for wild type *fd* viruses^46^. Between *T*_2_ and *T*_1_ in the isotropic droplets, 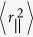 and 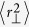 are similar confirming the isotropic nature of the droplets. In the case of the orientational mean square displacements 〈*θ*^2^〉, we observe a different behavior. At short time scales, 〈*θ*^2^〉 always vary linearly with time: viruses exhibit orientational diffusion. At intermediate time scales, we observe the appearance of a plateau in the nematic state: each individual rod is orientationaly “caged” along the director by its neighbour particles. The maximal tilt angle between long axis of virus and the director is described by the plateau value *θ*_*M*_. *θ*_*M*_ increases as temperature increases and the viruses explore a wider range of orientations around the director. Above *T*_2_, the plateau vanishes and the viruses undergo free rotational diffusion at all time scales.

**Figure 6:**
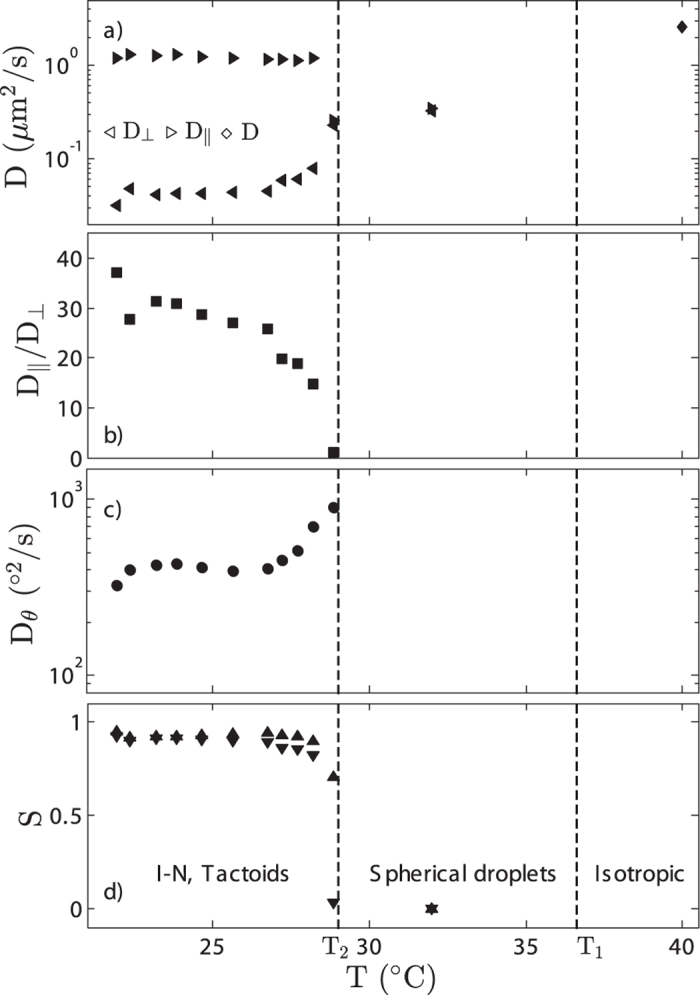
Evolution of the diffusion coefficients and the order parameter as a function of temperature during the *I* − *N* transition. (**a**) Short–time translational diffusion coefficients along *D*_||_ and perpendicular *D*_⊥_ to the director of the tactoid. *D* is the diffusion coefficient of the rods in the isotropic state. (**b**) The ratio of the diffusion coefficient parallel and perpendicular to the director. (**c**) Orientational diffusion coefficients *D*_*θ*_. (**d**) Order parameter *S* within the nematic droplets calculated using eq. 2 with the diffusion coefficient (▼) and with *θ*_*M*_ (▲).

The short–time diffusion coefficients calculated on the basis of the trajectory are presented in Fig. 6a–c and qualitatively agree with the theory of translational diffusion in nematic liquid crystals which is developed from the standpoint of hydrodynamics and molecular–hydrodynamic interactions^47^. *D*_||_ does not vary with temperature until it drops by a factor ~5 in the vicinity of *T*_2_. In contrast *D*_⊥_ and *D*_*θ*_ are constant far away from *T*_2_ but increase close to *T*_2_. High values of *D*_||_/*D*_⊥_ far from *T*_2_ are reasonable due to the liquid crystalline ordering and the large aspect ratio of the viruses which both favour a motion along the director rather perpendicular to the director. For comparison, *D*_||_/*D*_⊥_ for *fd* viruses in the nematic phase coexisting with isotropic phase ≈7.5^46^. Smaller values of *D*_||_/*D*_⊥_ obtained for fd–wild type viruses results from their smaller aspect ratio (*AR* = 133 instead of 183 for M13K07). Such tendency is confirmed by simulations of brownian dynamics of sphero–cylinders^48^. Decrease of *D*_||_/*D*_⊥_ as a function of temperature is related to a decrease of the aspect ratio of the nematic droplets as they become shorter and wider. Change of the droplets shape close to *T*_2_ indeed widens the space for perpendicular and rotational motion.

The order parameter is calculated using two independent approaches. On one hand, the translational diffusion coefficients are correlated with the nematic order parameter *S*, eq. 2,^48^. And on the other hand, it is possible to obtain *S* strictly from its definition using *θ*_*M*_ the maximal tilt angle between the long axis of viruses and the nematic director, eq. 2,^49^.


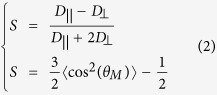


As shown in Fig. 6c both measurements of *S* are similar although there is a slight difference just before *T*_2_. Compared to typical thermotropic molecular liquid crystal, we observe much higher values of *S* even in the vicinity of the phase transition. Again, it results from the large aspect ratio of the viruses investigated. Both, simulations and experiments^50^,^51^,^52^,^53^ demonstrate that the nematic order parameter of rod–like molecules in coexistence with isotropic phase grows with increasing aspect ratio (*AR*) of the molecules and reaches 0.842 for *AR* = 50. The aspect ratio of M13K07 viruses is equal to *AR* = 183 but since viruses are semi–flexible, the order parameter at the nematic–isotropic transition is slightly lower than expected (*S* = 0.82 and *S* = 0.90 for calculation based on diffusion coefficients and *θ*_*M*_, respectively).

## Conclusion and Perspectives

In conclusion, on the engineering side, our system provides a new tool to navigate continuously in the phase diagram of colloidal rod–like particles, thanks to the pnipam microgel particles that mediate a temperature tunable attraction between the rods. On the physical side, we report the first optical microscopy imaging step by step of the condensation and dissolution of nematic droplets. We have shown that the condensation of nematic droplets from the isotropic state fallows a peculiar route: nucleation and growth of dense isotropic spherical droplets within the isotropic background then morphogenesis of the spherical droplets into tactoids. This route shows some analogy with the enhanced protein crystallization slot just above liquid–liquid phase separation suggested by ten Wolde and Frenkel^54^. Just as the critical density fluctuations and in particular fluctuations of high densities act as a micro reactor to lower the energy barrier for crystal nucleation, the isotropic dense droplets form the ideal nucleation spot for the nematic phase. Finally local measurements of the motion of individual rods confirm the role of the rotational diffusion as a driving force of the Isotropic–Nematic transition as predicted by Onsager. By tracking individual viruses, we have in particular shown, that the rods orientation is limited around the director to an average amplitude *θ*_*M*_ which value allows us to calculate the order parameter and characterize the isotropic-nematic transition.

**Figure 7:**
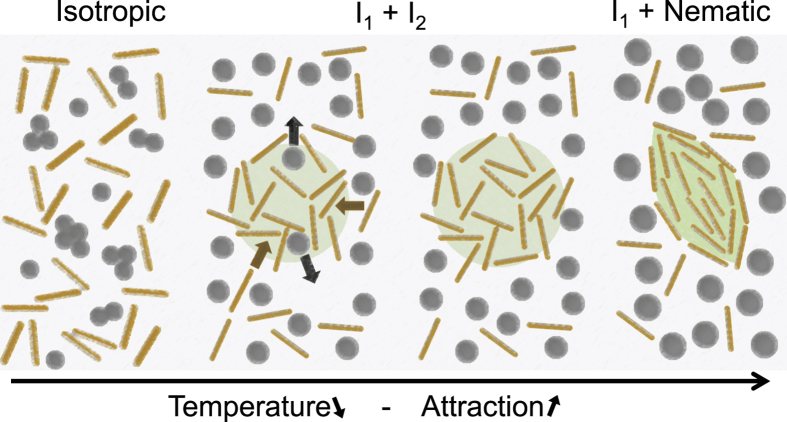
Schematic interpretation of the tactoid condensation. In the isotropic–isotropic coexistance regime (*I*_1_ − *I*_2_), where dense droplet of composition *I*_2_ coexist with the background dilute isotropic phase *I*_1_, rods would be concentrated in the droplet and microgel particles would be expelled from the droplet.

Many experiments show tactoid formation^16^,^18^,^21^,^46^ but none show isotropic dense droplets as a pathway to tactoids formation. We speculate that this discrepancy is due to the presence of attraction which promote liquid-liquid phase separation, and the large size of the depletant. The ability of the depletant to occupy the free space or to be expulsed form the ordered colloidal phase seems to be a key kinetic parameters in colloidal condensation as shown in the depletion induced crystallization of cubic colloids^39^. In our system, we believe that, first, microgel particles coexist with the viruses within the droplet and concentrate the viruses but prevent nematic order. The nematic phase does not contain depletants^31^ and would then form as the result of the expulsion of the microgel particles from the droplets, Fig. 7. Lekkekerker *et al.* works hint toward this suggestion. They have shown the existence of the isotropic–isotropic (*I*_1_ − *I*_2_) coexistence at lower attraction than the *I* − *N* coexistence in dispersions of rods with depletion but only with rods having a small aspect ratio^17^,^37^. On the simulation side, S. I. Hernández *et al.*^55^ show that liquid crystal confined in spherical nano-droplet display a variety of structure, and, in particular, may encapsulate an isotropic state or a nematic state. R. Berardi *et al.*^56^ assess how temperature controls the dynamics of droplets formation in dispersion of attractive rods. Their results match closely our observations although they observe rather than droplets, aggregates with very few rods.

Finally, we trust that we have unveiled an interesting pathway to tune the shape and the inside order of liquid crystal droplets which are of considerable importance for composites that act as light modulators or more generally as photonic materials^12^,^57^,^58^,^59^.

## Methods

### M13K07 viruses

M13K07 viruses are synthesized using standard biological protocols^33^. After synthesis, we usually get small portions of viruses that bind to each other through their end–to–end cap proteins and form virus dimers and trimers. We fractionated the viruses through the isotropic–nematic phase transition^1^. Only the isotropic fraction, enriched in nominal length viruses is kept for this work. These highly monodisperse viruses are dispersed at c_*V*_ = 0.3 mg/mL in 20 mM TRIS buffer at pH = 8.0 and 100 mM NaCl to screen the electrostatic charges. In order to observe trajectories of individual rods, a small amount of fluorescently labelled viruses is added to the dispersion. Fluorescent viruses are obtained by labelling the primary amines of the major coat protein of M13K07 with amine reactive fluorophore (DyLight–NHS ester 550, Thermo Fisher). The ratio of labelled to unlabelled rods is 1:1000.

### Pnipam microgel particles

Thermo–responsive and non–adsorbing pnipam microgel particles are synthesized according to a standard protocol^34^. First, 45 ml of deionized water, 25 mg of the cross linker BIS, N,N’-Methylenebisacrylamide, (Polysciences, Inc.), 75 mg acrylic acid and 2.5 mg of NIPA, N-isopropylacrylamide, (Polysciences, Inc.), are loaded into a special three-neck flask equipped with a stirrer, thermometer, and a gas inlet. The resultant mixture is stirred, heated to 78 °C, and bubbled with dry nitrogen for 10 minutes to remove dissolved oxygen. A solution of 25 mg of ammonium persulfate dissolved in 5 ml of deionized water is then added to the mixture to start the polymerization reaction. The mixture is continuously stirred at 78 °C for 30 minutes and then allowed to cool down to room temperature. Finally the particles are cleaned cyclically, first concentrating them by centrifugation and then re-suspending them in deionized water. The process is repeated approximately five times. Dynamic light scattering (DLS) measurements of pnipam microgel diluted in buffer to a concentration of 0.3 mg/ml are performed with the Malvern Zetasizer Nano Series. Using a cumulant analysis to fit the scattered intensity auto–correlation function^60^, we obtain the hydrodynamic radius as a function of temperature, Fig. 1b. Aqueous dispersions of pnipam polymers are well known to show a reversible lower critical solution temperature phase transition (LCST). When heated above 32 °C, the pnipam polymers undergoe a transition from a swollen hydrated state to a shrunken dehydrated state. The pnipam microgel behaves similarly. The hydrodynamic radius decreases slowly up to *T* = 26 °C and then more consequently up to *T* = 33 °C. Above *T* = 33 °C, the hydrodynamic radius increases and the solution becomes slightly turbid. At high salt concentrations, such the one we use, 100mM NaCl, pnipam microgel particles aggregate.

### Microscopy optical chambers

Samples are placed in optical chambers for microscopy experiments. The chamber is made from a microscope slide and a cover slip (Goldseal, Fisher Scientific) separated by a spacer consisting of melted Parafilm and sealed using ultraviolet–cured glue (Norland Optical). Glass surfaces are thoroughly cleaned with a hot 0.5% Hellmanex solution (Hellma Analytics) and coated with acrylamide brushes which prevent binding of the viruses to the glass surface^61^.

### Microscopy

The isotropic–nematic (*I* − *N*) phase transition is visualized using cross–polarization, phase contrast and fluorescence microscopy with the inverted microscope Nikon Eclipse T *i* equipped with an oil immersion objective (1.3 NA, 100x Plan–Fluor for cross–polarization and fluorecence microscopy and phase contrast microscopy) and with the high sensitivity CMOS camera Hamamatsu flash3 (1 pixel represents 64.5 nm at 100x). For the fluorescence we used the same setup with Mercury–Halide epi–fluorescence source and a rhodamine filter cube (excitation wavelength 532/554 nm, emission wavelength 570/613 nm). The exposure time for acquisition of fluorescent images is 20 ms. Binning of 4 is used to gain sensitivity.

### Temperature control

Observations are made as a function of temperature for three different rates of heating and cooling (~0.5, 1.1 and 3.1 °C/min, see Supplementary Fig. 1. The results are similar upon heating or cooling and independent of the rate. Samples are heated through the objective equipped with copper ring, located just below the specimen, and whose temperature is controlled with high precision (0.1 °C) by a thermostat (HUBER Ministat). The temperature inside the optical cell is measured with a thermocouple. We use this temperature as a reference because there is usually a few degrees difference with the temperature indicated by the thermostat and the thermocouple.

### Image analysis

The shape of the droplet is first measured with an ellipsoid recognition algorithm, then the ellipsoid shape is corrected to a tactoid shape and double checked manually. We use a simple particle tracking algorithm to determine the position of fluorescently labeled viruses^62^,^63^. This algorithm is designed for localization and tracking of objects in a series of 2D images with a sub-pixel precision. By using radial–symmetry–based fitting we obtain the viruses center of mass coordinates with a precision of 70 nm. The *θ* angles between long axis of labeled viruses and the macroscopic director of the tactoids are measured with a precision of 4° by fitting individual viruses with an ellipsoid (regionprops function in Matlab). All measurements are performed at least 10 times to check for reproducibility and then averaged. We measure the translational mean square displacements along and perpendicular to the tactoid director, 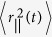 and 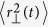 respectively, as well as orientation mean square displacement with respect to the director, 〈*θ*^2^(*t*)〉. Next, the diffusion coefficients *D*_||_, *D*_⊥_ and D_*θ*_ are determined from the slope of the mean square displacements versus time using following relations^48^,^50^:


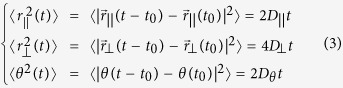


where 〈...〉 is an average over all rods and all *t*_0_.

## Additional Information

**How to cite this article**: Modlińska, A. *et al.* Condensation and dissolution of nematic droplets in dispersions of colloidal rods with thermo–sensitive depletants. *Sci. Rep.* **5**, 18432; doi: 10.1038/srep18432 (2015).

## Supplementary Material

Supplementary Information

Supplementary Movie 1

Supplementary Movie 2

Supplementary Movie 3

Supplementary Movie 4
