# Supplementary material for: Condensation and dissolution of nematic droplets in dispersions of colloidal rods with thermo–sensitive depletants
Source: Sci Rep. 2015 Dec 14;5:18432. doi: 10.1038/srep18432 (PMC4995677; doi:10.1038/srep18432)
Supplement: Supplementary Information [file srep18432-s1.pdf]

Condensation and dissolution of nematic droplets in dispersions of colloidal rods with thermo–sensitive depletants

Anna Modlińska[1,2], Ahmed M. Alsayed[3], and Thomas Gibaud[1,*]

[1]Labaroire de physique, CNRS/UMR 5672, Ecole Normale Supérieure de Lyon – Université de Lyon, 46 allée d'Italie, 69007 Lyon, France
[2]Faculty of Technical Physics, Poznan University of Technology, ul. Piotrowo 3, 60-965 Poznań, Poland
[3]Complex Assemblies of Soft Matter (COMPASS), Solvay-CNRS-UPenn UMI 3254, Bristol, Pennsylvania 19007, USA

ABSTRACT

Supplementary materials

1 Supplementary movies

MOVIE 1. Phase contrast imaging of a temperature quench from T=40°C to 22°C of the aqueous dispersion of rods and pnipam microgel particles. Quench rate 0.5°C/min. Image size 132x132 μm^2.

MOVIE 2. Phase contrast imaging of a temperature quench from T=22°C to 40°C of the aqueous dispersion of rods and pnipam microgel particles. Quench rate 1.1°C/min. Image size 132x132 μm^2.

MOVIE 3. Fluorescence imaging of labelled viruses within the spherical droplet. Image size 25.8x25.8 μm^2. Duration 10 s.

MOVIE 4. Fluorescence imaging of labelled viruses within the tactoid. Image size 51.6x103.2 μm^2. Duration 10 s.

2 Supplementary figures

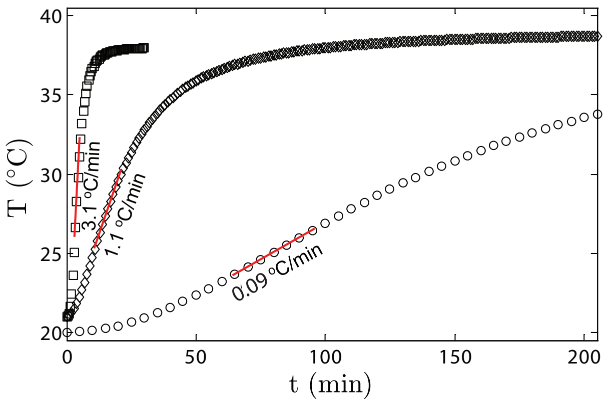

Figure 1. Three different heating rates used to quench the samples: 3.1, 1.1 and 0.09°C/min. The temperature is measured inside the optical cell with a thermocouple.

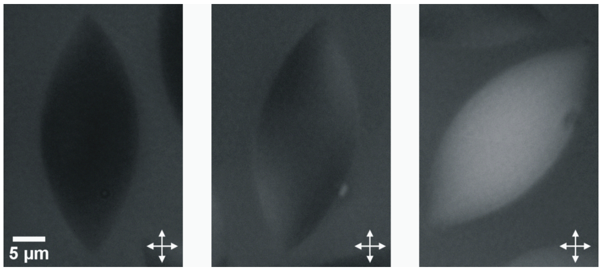

Figure 2. Textures of the tactoids observed under crossed polarizers with different angles of polarizers rotation with respect to the main axis of the tactoid.
